# Disease control and quality of life in chronic spontaneous urticaria and recurrent angioedema are strongly linked, but not in all patients

**DOI:** 10.1002/clt2.70026

**Published:** 2025-01-03

**Authors:** Sophia Neisinger, Pascale Salameh, Annika Gutsche, Felix Aulenbacher, Frank Siebenhaar, Marcus Maurer

**Affiliations:** ^1^ Institute of Allergology Charité – Universitätsmedizin Berlin Corporate Member of Freie Universität Berlin and Humboldt‐Universität zu Berlin Berlin Germany; ^2^ Fraunhofer Institute for Translational Medicine and Pharmacology ITMP Immunology and Allergology Berlin Germany; ^3^ School of Medicine Lebanese American University Byblos Lebanon; ^4^ Institut National de Santé Publique d'Épidémiologie Clinique et de Toxicologie‐Liban (INSPECT‐LB) Beirut Lebanon; ^5^ Department of Primary Care and Population Health University of Nicosia Medical School Nicosia Cyprus

**Keywords:** angioedema, chronic spontaneous urticaria, patient‐reported outcome measure

## Abstract

**Background:**

Patient‐reported outcome measures (PROMs) help to assess disease control and quality of life (QoL) in chronic spontaneous urticaria (CSU) and recurrent angioedema (RA). This study aimed to assess the correlation between two different concepts: disease control and QoL, using disease‐specific PROMs.

**Methods:**

We analyzed data from 445 CSU and 330 RA patients who completed both a disease control and QoL PROM as part of the clinical routine. We included the UCT and CU‐Q2oL for CSU and AECT and AE‐QoL for RA.

**Results:**

In CSU and RA, disease control scores positively correlated with QoL scores (Spearman's rho correlation coefficient (CR) −0.757, −0.735; *p* < 0.001) with better disease control corresponding to better quality of life. However, 5.9% of CSU patients and 28% of RA patients with complete disease control had impaired QoL. In CSU, QoL was impaired in 69.2% of patients based on the CU‐Q2oL and in 62.7% of patients based on a single numeric question from the UCT, with a mismatch in 89/445 patients. In RA, QoL was impaired in 58.5% using the AE‐QoL and in 52.7% using a single numeric question from the AECT30mo, with a mismatch in 69/330 patients. Different domains of the QoL PROMs showed different degrees of influence on disease control, with “Itching/Embarrassment” showing the strongest correlation with the UCT (CR −0.804; *p* < 0.001) and “Functioning” with the AECT3mo (CR −0.824; *p* < 0.001).

**Conclusion:**

Although most patients with controlled disease have better quality of life, unexpectedly, quality of life remains impaired in up to one‐fourth of patients with completely controlled CSU and RA. Reasons behind this should be investigated in further studies.

## 1 INTRODUCTION

Chronic urticaria and recurrent angioedema are chronic skin disorders that share pathogenic pathways, that is, mast cell degranulation, although not all forms of recurrent angioedema are mast cell driven.^1^ Chronic urticaria is characterized by itchy wheals and/or angioedema, symptoms that occur spontaneously (chronic spontaneous urticaria; CSU) or that can be induced by different kinds of triggers (chronic inducible urticaria; CindU).^2^ Recurrent angioedema (RA) is characterized by deeper swellings in the subcutis. In most patients, RA is due to mast cell activation, but can also be due to bradykinin and other mechanisms, for example, in hereditary angioedema.^3^ Chronic urticaria patients, especially those who also have angioedema, often suffer from quality of life impairment,^4^ for example, due to severe pruritus and the unpredictability of the symptom occurrence.^5^, ^6^, ^7^

The international urticaria guideline^2^ and the WAO/EAACI guideline for hereditary angioedema^8^ explicitly recommend the use of Patient‐Reported Outcome Measures (PROMs) to assess disease control and quality of life. Disease control and quality of life are two different concepts, and both are important when assessing treatment needs and efficacy.^2^, ^9^ Disease activity, control and impact in chronic inflammatory diseases are linked,^10^, ^11^ but there is little information for CSU and RA on how disease control is linked to QoL.

To address this gap of knowledge, we analyzed whether disease control is linked to QoL impairment in CSU and RA, making use of validated PROMs. We investigated how disease control affects different levels of quality of life in patients with CSU and RA by examining quality of life scores among patients with poor, well, and complete disease control. Moreover, we assessed the correlation between disease control and QoL as evaluated by a single global patient assessment question. We compared these findings with comprehensive QoL PROMs to determine the validity of using a single question for QoL assessment. Lastly, the study aimed to explore how disease control is associated with distinct domains of QoL in patients with CSU and RA to provide a detailed understanding of how different aspects of quality of life are impacted by disease control.

## 2 METHODS

### 2.1 Study design and participants

This was an observational, retrospective study that analyzed data of patients with CSU and RA, who visited the Urticaria and Angioedema Center of Reference and Excellence (UCARE and ACARE) of the Institute of Allergology, Charité—Universitätsmedizin Berlin from September 2018 to July 2023. Patients completed Patient‐Reported Outcome Measures (PROMs) relevant to their condition as part of the routine clinical monitoring. A comprehensive dataset, with both the control and quality of life PROM scores, was compiled for a total of 445 patients with CSU and 330 patients with RA. Patients who completed only the control PROM or the QoL PROM were excluded. The study has been approved by the local ethics committee (reference number: EA1/048/24).

### 2.2 Instruments

Patient‐Reported Outcome Measures (PROMs) are validated and structured questionnaires are used to assess disease activity, disease control, and quality of life impairment.^12^ The Urticaria Control Test (UCT) is a validated PROM designed to determine the level of disease control in individuals with CSU and CIndU. It has four questions, with a recall period of four weeks, leading to a total score range of 0–16 points. While 16 points indicate total disease control, 12–15 points indicate good and 0–11 poor disease control.^13^, ^14^ The Chronic Urticaria Quality of Life Questionnaire (CU‐Q2oL), with a two week recall period, is a validated 23‐item PROM to measure the quality of life impairment in patients with CSU. The score ranges from 0 to 100 (normalized to 100), with >20 indicating impairment in the quality of life of the patient.^15^, ^16^, ^17^ The CU‐Q2oL is divided into six domains, assessing different aspects of the quality of life of chronic urticaria patients: functioning, sleep, itching/embarrassment, mental status, swelling/eating and limits looks.^18^ Patients with chronic spontaneous urticaria completed the UCT and the CU‐Q2oL.

The AECT3mo compromises four questions, scoring from 0 to a maximum of 16 points, where a score of 10 or higher indicates good disease control, and 16 total disease control of patients with recurrent angioedema.^19^ To assess the quality of life impairment of patients with angioedema, the 17‐item AE‐QoL with a four week recall period can be used.^20^ The score ranges from 0 to 100, with a score of 30–39 indicating mild, 40–49 moderate and above 50 severe quality of life impairment. It is calculated by assigning points to each response (Never: 0, Rarely: 1, Sometimes: 2, Often: 3, Very often: 4), summing the points for all questions, and dividing by the maximum possible score (17 × 4) to obtain a percentage value. The AE‐QoL includes four domains: functioning, fatigue/mood, fears/shame and food.^20^, ^21^ Patients with recurrent angioedema completed the AECT3mo and the AE‐QoL, both applicable for patients with mast cell mediated, bradykinin mediated, or idiopathic angioedema.^2^, ^8^ The AECT can be used with a 4 week (AECT) or a 3 month recall period (AECT3mo).^22^, ^23^ We chose to include the AECT with a 3 month recall period (AECT3mo) because of a larger number of complete PROM data sets.

### 2.3 Statistics

We applied Spearman's Rho Correlation Coefficient to assess the relationship between control test outcomes and quality of life scores for each disease. The Kruskal‐Wallis Test was performed to compare quality of life scores across various control categories. For evaluating differences in Qol scores between the two control categories, the Mann‐Whitney *U* Test was used, given the non‐normal distribution of our continuous or ordinal dependent variables.

To compare mean quality of life scores across different control categories in CSU and recurrent angioedema, ANOVA (Analysis of Variance) was conducted when its assumptions were met (or the Kruskal Wallis non‐parametric counterpart when assumptions were not met). Subsequently, Post Hoc Tests, including Bonferroni or Tamhane methods, were applied to identify specific group differences in QoL scores following significant findings when comparing means between groups for each condition. Moreover, Kappa coefficients were used to determine the concordance between qualitative variables measuring similar concepts. Statistical significance was defined as *p* < 0.05.

Statistical analysis was performed using the Statistical Package for Social Sciences (SPSS) for Windows Version 28.0 (SPSS Inc).

## 3 RESULTS

### 3.1 In CSU and RA, high levels of disease control are linked to low disease impact on quality of life

**FIGURE 1:**
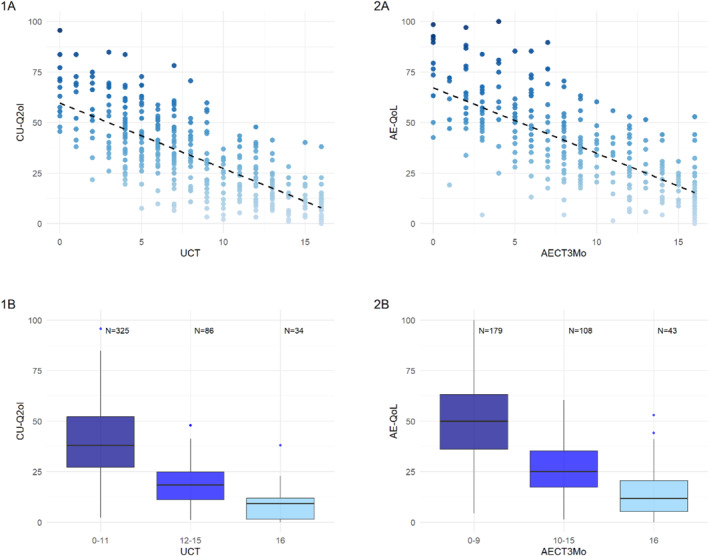
1(A): Mean CU‐Q2oL score among UCT, 1(B): Mean CU‐Q2oL score among UCT control groups, 2(A): Mean AE‐QoL score among AECT3mo, 2(B): Mean AE‐QoL score among AECT3mo control groups. *+ = not impaired QoL, ‐ = impaired QoL.

In 445 patients with CSU, UCT values and CU‐Q2oL scores showed a high correlation, −0.757 (*p* < 0.001; Figure 1). CSU patients with poor, well, and complete disease control showed statistically significant differences in CU‐Q2oL scores (all *p* < 0.001). Specifically, patients with poor disease control (UCT: 0–11) had a mean CU‐Q2oL score significantly higher (indicating worse QoL) than those with well controlled CSU (UCT: 12–15) or complete control (UCT: 16), with mean scores of 37.1 (SD = 16.7), 18 (SD = 10.1), and 7.7 (SD = 7.5) respectively (Figure 1).

For RA, disease control and impact on QoL, assessed in 330 patients by AECT3mo and AE‐QoL, were closely linked (CR −0.735, *p* < 0.001; Figure 1). RA patients with poorly, well, and completely controlled disease showed different QoL impairment, where patients with poor disease control (AECT: 0–9), good control (AECT: 10–15), and complete control (AECT: 16) had AE‐QoL scores of 50.5 (SD = 20), 26.8 (SD = 13.4), and 14.3 (SD = 12.4), respectively (Figure 1).

Across the two diseases, the link of disease control and QoL impairments showed different strengths, with a Kappa concordance coefficient for inter‐rater reliability, a measure of how consistently the levels of disease control match the corresponding QoL impairment, of 0.381 (*p* < 0.001) and 0.305 (*p* < 0.001) in CSU and RA, respectively.

### 3.2 Quality of life is impaired in up to one‐fourth of patients with completely controlled chronic spontaneous urticaria and recurrent angioedema

Among patients with completely controlled CSU, 5.9% had impaired QoL, that is, a CU‐Q2oL score of >20. In patients with well‐controlled CSU, 37.2% experienced QoL impairment.

For angioedema patients with complete disease control (AECT: 16), 14%, 7%, and 7% had mild, moderate, and severe QoL impairment, respectively. In patients with well controlled disease (AECT: 10–15), QoL was impaired in 66.7% (mild: 23.1%, moderate: 15.7%, severe QoL impairment: 27.8%).

### 3.3 For measuring QoL in patients with CSU or RA, the use of dedicated PROMs cannot be replaced by a single question global patient assessment

**FIGURE 2:**
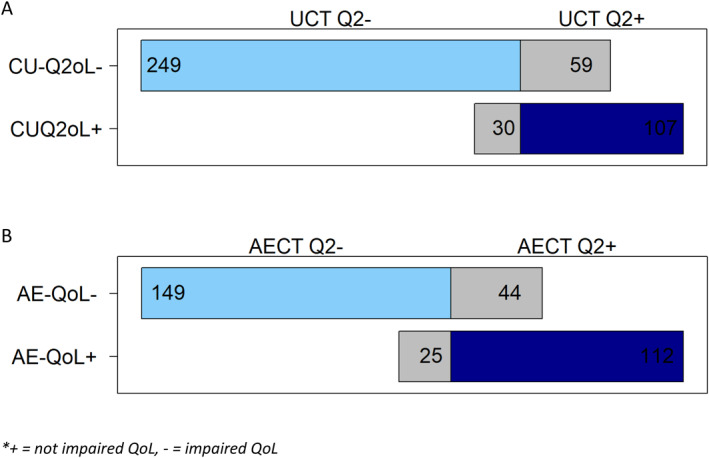
(A): quality of life of CSU patients assessed with the UCT Question 2 (UCT Q2) and the CU‐Q2oL (B): quality of life of RA patients assessed with the AECT Question 2 (AECT Q2) and the AE‐QoL.

QoL impairment, in CSU, determined with a single question global patient assessment, that is, question 2 of the UCT (“How much was your quality of life affected by the urticaria in the last 4 weeks?”), showed a strong correlation with the CU‐Q2oL (CR −0.745, *p* < 0.001; Table 1). However, the outcomes mismatched in 89 of 445 patients (20%; Figure 2). QoL, based on the assessment with a single question, was impaired in 279 patients (62.7%), with 61 (21.9%), 96 (34.4%), and 122 (43.7%) of patients reporting that their QoL was impaired very much, much, and somewhat, respectively. QoL, based on the CU‐Q2oL was impaired in 69.2% (308/445 patients).

**TABLE 1:** Spearman's rho correlation (CR) between UCT question 1–4 and CU‐Q2oL; Spearman's rho correlation (CR) between AECT3mo question 1–4 and AE‐QoL.

UCT	*N*	CR (CU‐Q2oL)	AECT3mo	*N*	CR (AE‐QoL)
Question 1 UCT	445	−0.689^a^	Question 1 AECT	330	−0.654^a^
Question 2 UCT	445	−0.745^a^	Question 2 AECT	330	−0.732^a^
Question 3 UCT	445	−0.560^a^	Question 3 AECT	330	−0.713^a^
Question 4 UCT	445	−0.680^a^	Question 4 AECT	330	−0.531^a^

^a^ Correlation is significant at the 0.01 level (2‐tailed).

For RA, QoL impairment based on question two of the AECT3mo (“In the last 3 months, how much has your quality of life been affected by angioedema?”) showed a strong correlation with QoL assessed with the AE‐QoL (CR −0.732, *p* < 0.001; Table 1). However, the outcomes mismatched in 69 of 330 patients (20.9%; Figure 2). QoL, based on the assessment with a single question, was impaired in 174 patients (52.7%), with 41 (23.6%), 57 (32.8%), and 76 (43.7%) of patients reporting that their QoL was impaired very much, much, and somewhat, respectively. QoL, based on the AE‐QoL was impaired in 58.5% (193/330 patients).

### 3.4 In CSU and RA, the correlation of disease control with QoL differs across domains

In patients with CSU, UCT scores showed the strongest correlation with CU‐Q2oL assessed impairment in the QoL domain “Itching/Embarrassment” (CR −0.804, *p* < 0.001), followed by “Functioning” (CR −0.765, *p* < 0.001), “Swelling/Eating” (CR −0.465, *p* < 0.001), “Sleep” (CR −0.459, *p* < 0.001), “Mental status” (CR −0.408, *p* < 0.001) and “Looks” (CR −0.367, *p* < 0.001) (Table 2).

**TABLE 2:** Spearman's rho correlation (CR) of UCT and CU‐Q2oL domains/of AECT3mo and AE‐QoL domains.

CU‐Q2oL domain	*N*	CR (UCT)
CU‐Q2oL functioning	445	−0.765^a^
CU‐Q2oL sleep	445	−0.459^a^
CU‐Q2oL itching/embarrassment	445	−0.804^a^
CU‐Q2oL mental status	445	−0.408^a^
CU‐Q2oL swelling/eating	445	−0.465^a^
CU‐Q2oL limits looks	445	−0.367^a^

AE‐QoL domain	*N*	CR (AECT)
AE‐QoL functioning	330	−0.824^a^
AE‐QoL fatigue/mood	330	−0.382^a^
AE‐QoL fears/shame	330	−0.612^a^
AE‐QoL food	330	−0.529^a^

^a^ Correlation is significant at the 0.01 level (2‐tailed).

In patients with RA, disease control measured with the AECT3mo showed the strongest correlation with AE‐QoL assessed impairment in the QoL domain “Functioning” (CR −0.824, *p* < 0.001), followed by “Fears/Shame” (CR −0.612, *p* < 0.001), “Food” (CR −0.529, *p* < 0.001) and “Fatigue/Mood” “(CR −0.382, *p* < 0.001)” (Table 2).

## 4 DISCUSSION

Our study provides a comprehensive analysis of the relationship between disease control and quality of life in chronic spontaneous urticaria and recurrent angioedema. It contributes to the literature by emphasizing the complexity of the relationship between disease control and quality of life.^10^, ^11^, ^24^ It challenges the assumption that better disease control always correlates with improved quality of life.

In both conditions, a significant correlation was observed between disease control and quality of life. This shows a trend that better disease management might be associated with improved quality of life. It is crucial to note that moving patients from good to complete disease control might be linked to better quality of life in chronic spontaneous urticaria and recurrent angioedema, as shown by our results. This observation provides additional support to the aim of complete disease control in these diseases.^25^ In clinical practice, this implies that even if a patient's condition is already well controlled, it might be beneficial for the patient’s QoL to adjust their therapy.

Interestingly, some patients with complete disease control still exhibit markedly impaired quality of life. This finding highlights that the correlation between control and QoL does not translate to all patients. Moreover, this shows that there are more factors that influence the quality of life of patients than disease control.^26^, ^27^ This reinforces the recommendation to assess patients for disease control and QoL rather than only one of them, and use the mentioned disease‐specific PROMs.^28^ Given that factors beyond disease control affect QoL, psychological support can be considered, as depression and anxiety are known to be associated with CSU and RA.^29^, ^30^ An interdisciplinary approach collaborating with colleagues from psychiatry/psychology could possibly benefit these patients. Understanding the potential causes of impaired QoL in patients could potentially help prevent the development of a psychological disorder. In addition, several studies have shown that in general patients with a chronic illness should get psychological support.^31^, ^32^

As expected, the quality of life question in the control PROM of both conditions showed a significant and strong correlation to the overall QoL PROM score. But it mismatched in 89 CSU and 69 RA patients, with the CU‐Q2oL and the AE‐QoL detecting more patients with QoL impairment than the single question global patient assessment. The CU‐Q2oL as well as the AE‐QoL, different from the UCT and AECT, have been extensively validated as a reliable measure for QoL in patients with chronic urticaria and angioedema, ensuring that they accurately reflect the QoL impact. Different from the single question from the control tests, the CU‐Q2oL and AE‐QoL provide a comprehensive assessment of QoL by covering a wide range of domains affected by the condition. Since the CU‐Q2oL and AE‐QoL are more complex and time‐consuming than the control tests, the questions on quality of life in the control PROMs, especially of the AECT, can give an idea on how QoL is impaired. However, this does not and should not replace the QoL PROMs, especially as the correlations were not complete, and these tests consider all relevant aspects of QoL, providing a more detailed assessment.

Subdomains of the quality of life PROMs might guide physicians toward unexpected directions and not only give information on the level of QoL impairment. In CSU, the strongest correlation was observed between the UCT and the “Itching/Embarrassment” domain of the CU‐Q2oL, highlighting that itching and embarrassment is particularly impactful. In the literature, itch is even referred to as the “skin equivalent of pain” by Kini et al.^33^ While it may not be common in clinical practice to ask patients if they for example, feel embarrassed about visiting public places, this aspect could be considered and might be important to know when seeing patients with comorbid psychiatric disorders.

In Angioedema, the “Functioning” domain showed the highest correlation with disease control, highlighting that functional impairments are a primary concern for these patients, especially those with poor disease control. This closer look at the AE‐QoL domains might help physicians in clinical practice to decide, for instance, if a hereditary angioedema (HAE) patient would benefit more from a long‐term prophylaxis^34^ or an on‐demand treatment, taking a deeper look into the patient's QoL impairment, as others might be bordered more by feeling ashamed of the symptoms. Building on these findings, the differences in QoL‐affected domains between CSU and RA emphasize that subdomains can provide nuanced insights into the distinct burdens of these conditions—with wheals and itch causing embarrassment for CSU patients, while RA patients experience greater functional impairments due to unpredictable and disabling swelling. Our results show that not only the overall QoL score should be used in clinical practice but also subdomains should also be taken into consideration for optimal treatment decisions.

A recent publication by Cherrez Ojeda et al. shows that PROMs for chronic urticaria are not frequently used by physicians, mostly due to a lack of time.^35^ This finding challenges our recommendation for physicians to take subdomains into consideration. The use of digital PROMs might be a solution here, as multiple studies have shown their positive effect mostly due to less time consumption, more completed questionnaires, and patient's preference^36^, ^37^. Digital PROMs can be completed on a tablet or on the patient's phone through an app, as demonstrated by the CRUSE app for CSU patients.^38^

A number of limitations may have influenced our reported results. We analyzed data from the specialized outpatient clinic of the Institute of Allergology, Charité‐Universitätsmedizin Berlin that might see a higher number of severe cases. It must be noted that other PROMs are available to assess disease activity, control and quality of life, in addition to those used in this study, many of which, such as the Dermatological Life Quality Index (DLQI), are not disease‐specific.^12^

Future studies with a larger sample size are needed to confirm our results and develop recommendations for managing patients with complete disease control but impaired QoL.

In conclusion, we showed that the relationship between disease control and quality of life in chronic spontaneous urticaria and recurrent angioedema is complex. Our findings underline, that both the control and quality of life PROMs should be used in clinical practice. A single question global patient assessment is not sufficient to assess quality of life impairment in CSU and RA. Notably, while achieving good control is beneficial, our results show that physicians should aim for full disease control, as it shows better quality of life results in these two conditions. If patients exhibit impaired QoL even with good or complete disease control, the QoL impairment should be further analyzed and psychological support could be considered.

## AUTHOR CONTRIBUTIONS

**Sophia Neisinger**: Conceptualization, methodology, formal analysis, data curation, project administration, visualization, investigation, writing—original draft, writing—review and editing, data curation; validation. **Pascale Salameh**: Methodology, formal analysis, data curation, writing—review and editing; validation. **Annika Gutsche**: Writing—review and editing; formal analysis. **Felix Aulenbacher**: Data curation, visualization, writing—review and editing. **Frank Siebenhaar**: Writing—review and editing; supervision; methodology; validation. **Marcus Maurer**: Supervision; methodology; conceptualization; writing—review and editing; validation.

## CONFLICT OF INTEREST STATEMENT

SN, PS, AG and FA have no conflicts of interest to declare. F. Siebenhaar received research funding from and/or is or recently was a speaker/advisor for Allakos, Blueprint, Celldex, Cogent, Escient, GSK, Granular, Invea, Moxie, Noucor, Novartis, Sanofi/Regeneron, Telios, ThirdHarmonicBio and UCB. Marcus Maurer was a speaker and/or advisor for and/or has received research funding from Allakos, Alexion, Alvotech, Almirall, Amgen, Aquestive, argenX, AstraZeneca, Celldex, Celltrion, Clinuvel, Escient, Evommune, Excellergy, GSK, Incyte, Jasper, Kashiv, Kyowa Kirin, Leo Pharma, Lilly, Menarini, Mitsubishi Tanabe Pharma, Moxie, Noucor, Novartis, Orion Biotechnology, Resoncance Medicine, Sanofi/Regeneron, Santa Ana Bio, Septerna, Servier, Third HarmonicBio, ValenzaBio, Vitalli Bio, Yuhan Corporation, Zurabio.
